# Effects of Exogenous Melatonin Concentration on Salt Stress Alleviation in *Salix matsudana* × *S. alba* Assessed Through Integrated Physiological Evaluation and Transcriptomic Analysis

**DOI:** 10.3390/plants15213304

**Published:** 2026-10-26

**Authors:** Zhenxiao Chen, Zhekai Deng, Hangze Han, Zhenan Chen, Hao Li, Handong Gao

**Affiliations:** 1 College of Forestry and Grassland, Nanjing Forestry University, Southern Tree Seed Inspection Center, National Forestry and Grassland Administration, Co-Innovation Center for Sustainable Forestry in Southern China, Nanjing 210037, China; 2 Jiangsu Academy of Forestry, Nanjing 211153, China

**Keywords:** melatonin, salt stress, principal component analysis, transcriptome, plant hormone signal transduction, sugar metabolism

## Abstract

Salt stress limits the establishment of *Salix matsudana* × *S. alba* seedlings. Exogenous melatonin (MT) can alleviate plant salt stress, but its concentration-dependent effects in *Salix matsudana* × *S. alba* remain insufficiently characterized. In this study, physiological responses were assessed across three NaCl levels and five MT concentrations, followed by transcriptome analysis of representative treatments. Under 6‰ NaCl, 0.5 mmol L^−1^ MT significantly reduced relative electrolyte conductivity and malondialdehyde content across three sampling times and increased total chlorophyll, proline, and soluble protein contents and antioxidant enzyme activities at the final sampling relative to the salt-only treatment. An integrated physiological assessment based on principal component analysis and supported by correlation analysis ranked 0.5 mmol L^−1^ highest under 6‰ NaCl, with 0.3 mmol L^−1^ ranking second, whereas 1.0 mmol L^−1^ generally provided no further overall improvement. Compared with the untreated control, the 6‰ NaCl plus 0.5 mmol L^−1^ MT treatment showed transcriptional changes involving plant hormone signal transduction and starch and sucrose metabolism. Quantitative reverse transcription PCR broadly supported RNA sequencing trends for nine candidate genes. These findings demonstrate concentration-dependent physiological protection and identify candidate pathways for investigating MT-associated salt stress alleviation in hybrid willow.

## 1. Introduction

Soil salinization threatens vegetation establishment and the long-term success of ecological restoration. The Food and Agriculture Organization of the United Nations estimates that approximately 1.381 billion ha, or 10.7% of global land area, are salt-affected [1]. Climate change may further increase salinization risks [2], while seawater intrusion and seasonal salt reaccumulation expose coastal vegetation to spatially heterogeneous and fluctuating salinity [3]. Excessive Na^+^ and Cl^−^ accumulation can directly damage cells and disrupt the uptake, transport, and compartmentation of essential elements such as K^+^ and Ca^2+^, thereby disturbing ionic and nutrient homeostasis [4]. Salt-induced redox imbalance can also cause excessive accumulation of reactive oxygen species (ROS), leading to membrane lipid peroxidation, protein damage, chlorophyll degradation, and reduced photosynthetic efficiency, ultimately limiting biomass production and accelerating tissue senescence [5]. Plants maintain homeostasis through ion exclusion and vacuolar compartmentation, osmotic adjustment, antioxidant defense, phytohormone signaling, and changes in stress-related gene expression [6,7], but the effectiveness of these responses depends on species, tissue, stress intensity, and duration [6]. Unlike short-lived herbaceous plants, perennial woody plants experience recurring salinity fluctuations across seasons. Their seedling salt tolerance often determines subsequent survival, stand stability, and ecological function, yet the regulation of salt tolerance in woody plants remains comparatively understudied [8]. Recent crop-oriented studies further emphasize that translating advances in salt-tolerance mechanisms and genes into stable performance under saline–alkaline field conditions remains an important challenge [9]. Selecting suitable salt-tolerant tree species and improving their stress adaptation are therefore important for afforestation, vegetation restoration, and sustained wind protection and soil stabilization on coastal saline–alkali land [10].

Willows (*Salix* spp.) are valuable for ecological engineering applications, including shelterbelt establishment, shoreline stabilization, degraded-soil remediation, and biomass utilization, because of their rapid growth, ease of vegetative propagation, extensive root systems, and high biomass production [11]. *Salix matsudana* × *S. alba*, a hybrid between *S. matsudana* and *S. alba*, shows potential adaptation to salinity and excess water. Research on material from the same hybrid combination has shown that biomass production and leaf condition depend not only on mean root-zone salinity but also on the vertical distribution of salts in the soil; root allocation among zones differing in salinity is an important component of adaptation to heterogeneous saline environments [12]. Under combined salt and waterlogging stress, *Salix matsudana* × *S. alba* can regulate genes associated with cell wall remodeling, osmotic adjustment, and ROS scavenging, together with Ca^2+^, plant hormones, and mitogen-activated protein kinase (MAPK) signaling, indicating transcriptional responses involving several pathways [13]. This adaptive potential can translate into ecological benefits: a five-year field study found that *Salix matsudana* × *S. alba* plantations increased organic matter and reduced electrical conductivity and total salt content in coastal saline–alkali soils, improving surface soil quality [14]. However, salt tolerance does not imply that plants maintain normal growth at every salinity level or stage of stress. Increasing salt stress can still reduce photosynthetic efficiency, chlorophyll levels, and membrane stability while increasing oxidative injury in *Salix matsudana* × *S. alba* [15]. Seasonal salt reaccumulation and interactions between salinity and waterlogging may further threaten seedling establishment in coastal areas. Although its environmental adaptability supports the use of this hybrid on saline land, effective external treatments are still needed to improve seedling salt tolerance and sustain its subsequent contribution to ecological restoration.

Melatonin (MT; N-acetyl-5-methoxytryptamine) is an indoleamine synthesized endogenously in plants and involved in redox regulation, signaling, and stress adaptation [16,17]. It can act as a non-enzymatic antioxidant by directly scavenging radicals, in addition to influencing enzymatic antioxidant systems [18,19]. Root-applied MT has been shown to reach maize leaves through transpiration-associated transport [20], and evidence for transporter-mediated uptake has been reported for tobacco NtMelT in Nicotiana benthamiana [21]. In the woody species *Malus hupehensis*, exogenous MT improves ion balance and antioxidant capacity, alleviating growth inhibition and maintaining chlorophyll and net photosynthetic rate [22]. A concentration-dependent response was also reported in Lonicera japonica, where an appropriate MT treatment improved vegetative growth, chlorophyll status, antioxidant enzyme activities, and the expression of stress-related genes under NaCl stress [23]. Beyond antioxidant protection, MT can regulate the accumulation of osmotic adjustment-related compounds, including proline (Pro) and soluble sugars (SS), linking water retention, carbon metabolism, and ROS scavenging [24]. Transcriptomic studies have implicated plant hormone signaling and starch and sucrose metabolism in alfalfa [25], and sugar metabolism-related genes and stress-responsive transcription factors (TFs) in banana seedlings [26]. More recently, integrated transcriptomic and metabolomic analyses in eggplant linked exogenous MT treatment under salt stress with coordinated physiological and molecular responses, including regulation of α-linolenic acid metabolism [27]. These findings support examining MT protection through integrated physiological and transcriptional responses rather than antioxidant effects alone. However, selecting an effective treatment requires more than demonstrating a beneficial response at a single concentration. Protective effects do not necessarily increase with concentration and also depend on application method, stress intensity, and plant material. A meta-analysis highlights the importance of these conditions for salt stress alleviation [28]. Although MT research in woody plants is expanding, mechanistic validation across biological levels remains limited [29]. For *Salix matsudana* × *S. alba*, it remains unclear which MT concentration provides relatively consistent physiological benefits across salt stress intensities and stages, and which transcriptional features accompany a favorable overall physiological state. Resolving these questions would connect concentration selection with evidence from multiple physiological traits and identify molecular processes for further investigation, providing a stronger basis for seedling stress management.

In this study, hybrid willow (*Salix matsudana* × *S. alba*) seedlings were subjected to three NaCl levels (0‰, 3‰, and 6‰, designated S1, S2, and S3, respectively) and five MT concentrations (0, 0.1, 0.3, 0.5, and 1.0 mmol L^−1^, designated M0–M4, respectively). This factorial design allowed MT concentration effects to be examined under both non-saline and saline conditions. Membrane injury, photosynthetic pigment status, osmotic adjustment, and antioxidant defense were assessed on days 20, 27, and 34. Accordingly, the physiological component of this study focused on leaf membrane injury, pigment status, osmotic adjustment-related compounds, and antioxidant defense; Na^+^, K^+^, and Ca^2+^ concentrations and related ion ratios in roots or leaves were not directly measured. Correlation analysis and principal component analysis (PCA) were used to examine relationships among the physiological traits and evaluate overall physiological condition. These analyses supported the assessment of MT concentration effects at each sampling time and the identification of treatments with relatively consistent protective effects. Representative treatments were then selected for transcriptome analysis, focusing on hormone signaling, carbohydrate metabolism, and stress-related regulatory responses. By integrating physiological screening with transcriptomic analysis of representative treatments, this study aimed to identify a favorable MT concentration and characterize transcriptional features associated with contrasting physiological states under salt stress. Linking treatment selection with these responses provides an experimental basis for further evaluation of MT in seedling production and saline-land restoration.

## 2. Results

### 2.1. Effects of Exogenous MT on Salt-Induced Membrane Injury and Chlorophyll Levels

**Figure 1:**
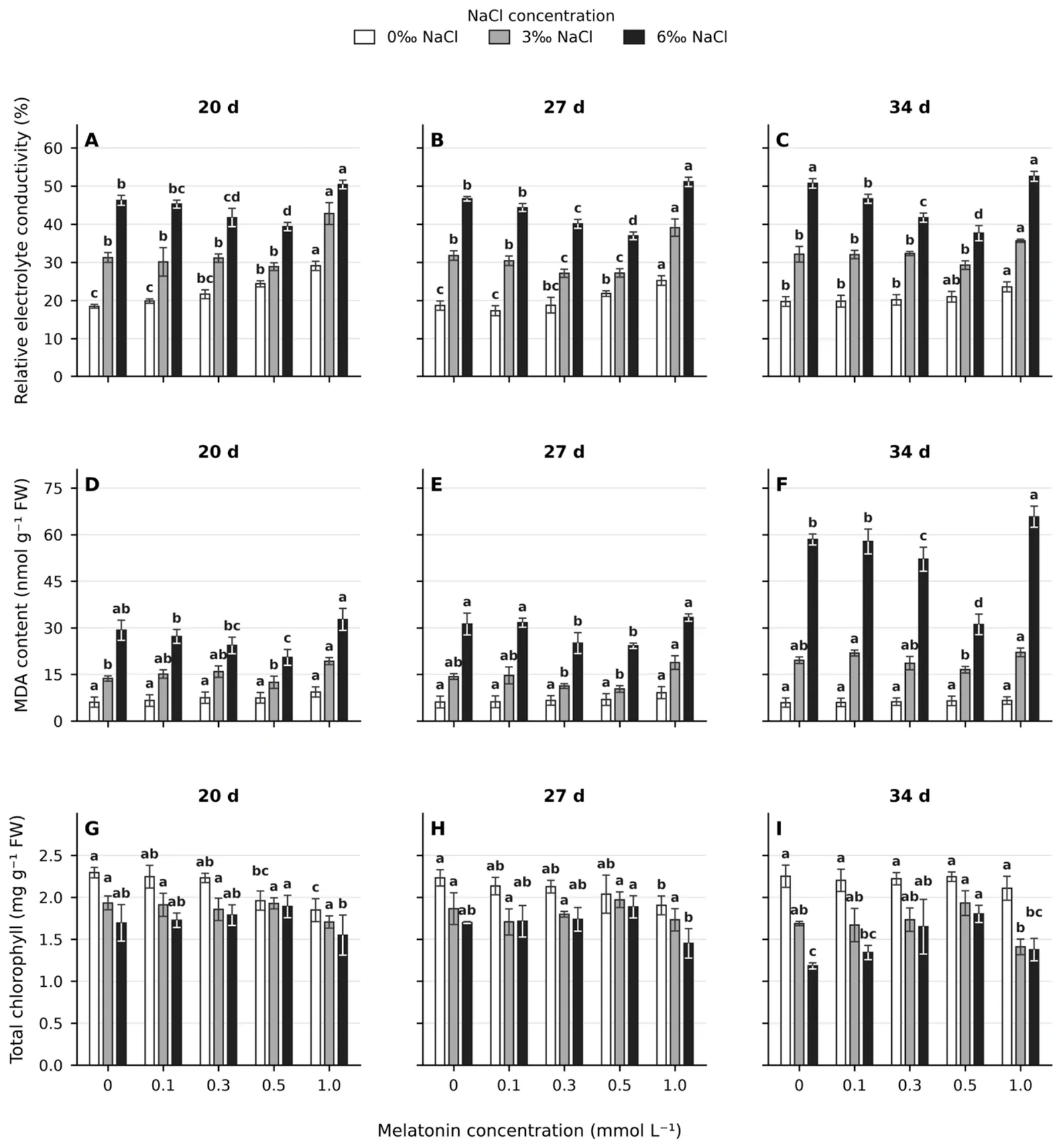
Effects of exogenous melatonin on membrane damage and chlorophyll content in leaves of *Salix matsudana* × *S. alba* seedlings under different NaCl concentrations. (**A**–**C**) Relative electrolyte conductivity; (**D**–**F**) malondialdehyde (MDA) content; (**G**–**I**) total chlorophyll content. Different lowercase letters indicate significant differences among MT concentrations within the same NaCl treatment and sampling time based on Tukey-adjusted pairwise comparisons (*p* < 0.05); bars sharing a letter do not differ significantly.

NaCl and MT treatments significantly affected leaf relative electrolyte conductivity (REC), malondialdehyde (MDA) content, and total chlorophyll (Chl) content in *Salix matsudana* × *S. alba* (Figure 1A–I). The full factorial repeated-measures analysis identified MT × time interactions for all eight physiological traits and NaCl × MT × time interactions for REC, MDA, Pro, SS, SP, SOD, and POD after BH correction across traits (q < 0.05; Table S1). The MT × time interaction for Chl was sensitive to the testing approach (Greenhouse–Geisser q = 0.0273; multivariate Pillai q = 0.0690) and was therefore interpreted cautiously.

The REC response to MT varied with NaCl level (Figure 1A–C). Under S3, REC was lowest in M3 at all three sampling times and was significantly lower than in M0. At the final sampling, REC was 37.65% in S3M3, compared with 50.72% in S3M0. Increasing the MT concentration to M4 resulted in significantly higher REC than that in M3 at all three sampling times. Under S2, the significant decrease in M3 relative to M0 occurred mainly on day 27, whereas M4 resulted in higher REC at all three sampling times. Under S1, M4 also significantly increased REC at each sampling time.

MDA broadly followed the pattern observed for REC (Figure 1D–F). Under S3, MDA content was significantly lower in M3 than in M0 at all three sampling times and was also lower in M3 than in M4. At the final sampling, MDA contents were 31.09 and 58.43 nmol g^−1^ FW in S3M3 and S3M0, respectively. Under S2, M3 generally had lower MDA, but the difference from M0 was not significant; MDA in M3 was significantly lower than in M4 at all three sampling times. Under S1, MDA did not differ significantly among MT treatments.

The Chl response to MT was most apparent at the later stage of severe salt stress (Figure 1G–I). Under S3, total chlorophyll content did not differ significantly between M3 and M0 at the first two sampling times. At the final sampling, Chl content was significantly higher in M2 and M3 than in M0 under S3. Chl content reached 1.80 mg g^−1^ FW in S3M3, compared with 1.18 mg g^−1^ FW in S3M0, whereas it did not differ significantly between M4 and M0. Under S2, Chl content did not differ significantly between M3 and M0, although it was significantly higher in M3 than in M4 at the final sampling. Under S1, Chl was significantly lower in both M3 and M4 than in M0 on day 20, whereas M4 was also significantly lower than M0 on day 27.

### 2.2. Effects of Exogenous MT on Leaf Osmotic Adjustment-Related Compounds and Antioxidant Enzyme Activities

**Figure 2:**
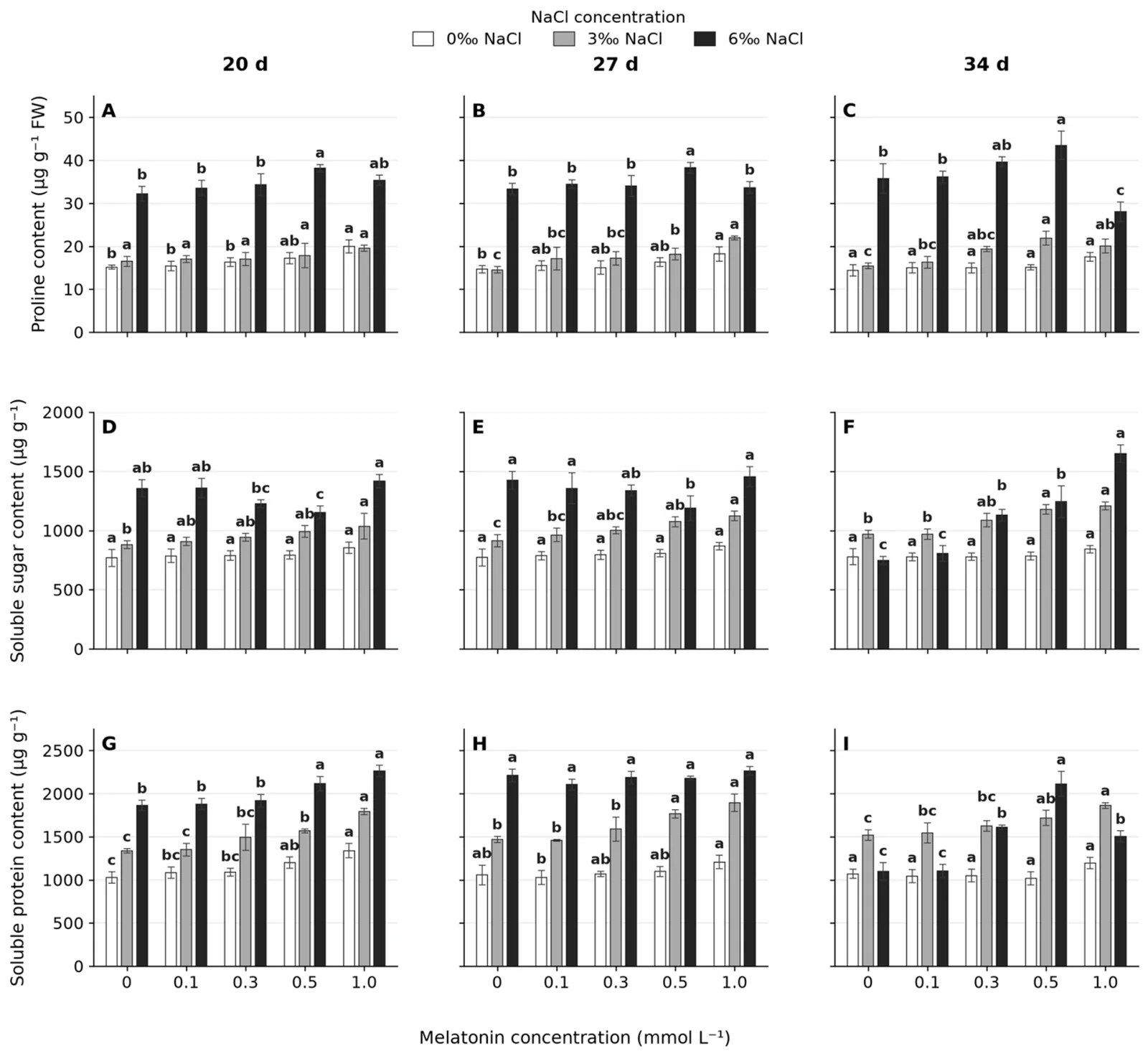
Effects of exogenous melatonin on osmotic adjustment-related compounds in leaves of *Salix matsudana* × *S. alba* seedlings under different NaCl concentrations. (**A**–**C**) Free proline (Pro) content; (**D**–**F**) soluble sugars (SS) content; (**G**–**I**) soluble protein (SP) content. Different lowercase letters indicate significant differences among MT concentrations within the same NaCl treatment and sampling time based on Tukey-adjusted pairwise comparisons (*p* < 0.05); bars sharing a letter do not differ significantly.

The response of leaf Pro to MT varied with salt stress intensity (Figure 2A–C). Under S3, M3 significantly increased Pro at all three sampling times, with a 21.6% increase relative to M0 at the final sampling. Increasing the MT concentration further to M4 instead decreased Pro, which was significantly lower than in M3. Under S2, differences among MT treatments were mainly evident at the final sampling, when both M3 and M4 were significantly higher than M0. Under S1, M4 significantly increased Pro relative to M0 on days 20 and 27, whereas the difference was not significant on day 34.

SS did not follow exactly the same pattern as Pro (Figure 2D–F). Under S3, SS content in M3 was significantly lower than in M0 on days 20 and 27. At the final sampling, SS content was significantly higher in M2–M4 than in M0. M4 had the highest SS content, which was significantly higher than that in M3. Under S2, higher MT concentrations were generally associated with higher SS on days 27 and 34, whereas no significant differences among MT treatments were observed under S1.

Differences in soluble protein (SP) among treatments were most apparent at the later stage of severe salt stress (Figure 2G–I). Under S3, M3 and M4 both significantly increased SP on day 20, whereas treatments did not differ significantly on day 27. At the final sampling, SP content was highest in M3 and was significantly higher than in M0 and M4. Under S2, SP content was significantly higher in M4 than in M0 at all three sampling times. Under S1, differences among MT treatments occurred mainly on day 20, with an additional significant difference between M4 and M1 on day 27.

**Figure 3:**
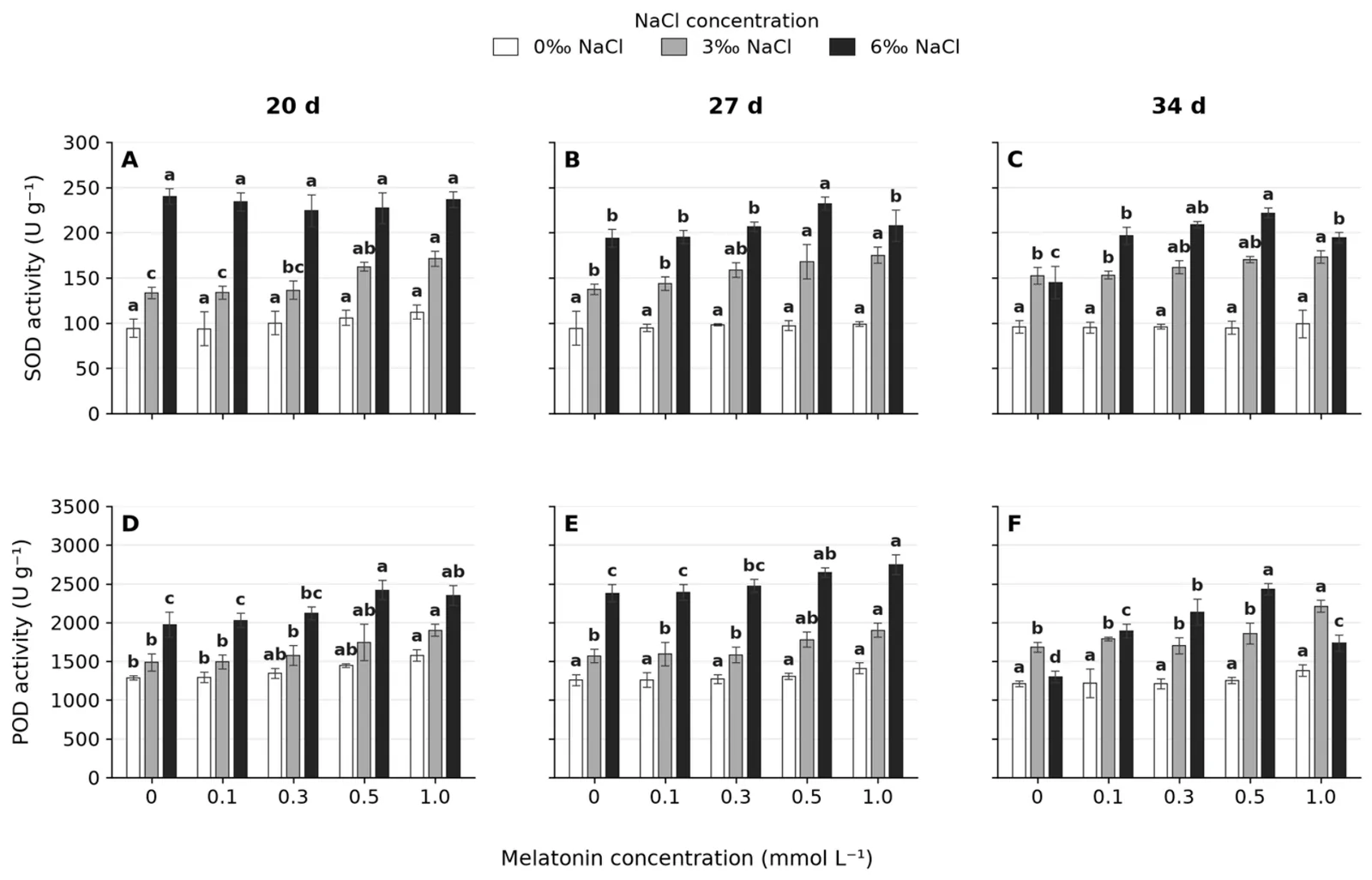
Effects of exogenous melatonin on antioxidant enzyme activities in leaves of *Salix matsudana* × *S. alba* seedlings under different NaCl concentrations. (**A**–**C**) Superoxide dismutase (SOD) activity; (**D**–**F**) peroxidase (POD) activity. Different lowercase letters indicate significant differences among MT concentrations within the same NaCl treatment and sampling time based on Tukey-adjusted pairwise comparisons (*p* < 0.05); bars sharing a letter do not differ significantly.

Superoxide dismutase (SOD) activity varied with MT concentration, NaCl level, and sampling time (Figure 3A–C). Under S3, MT treatments did not differ significantly on day 20, whereas SOD activity in M3 was significantly higher than in M0 on days 27 and 34. At the final sampling, SOD activity in M4 remained higher than in M0 but was significantly lower than in M3. Under S2, higher MT concentrations generally corresponded to higher SOD activity, whereas no significant differences among MT treatments were detected under S1.

Peroxidase (POD) showed similar variation with sampling time and NaCl level (Figure 3D–F). Under S3, POD activity was significantly higher in M3 than in M0 at all three sampling times, while M4 also showed relatively high activity on day 27. At the final sampling, POD activity in M3 was significantly higher than in M4. Under S2, POD activity was significantly higher in M4 than in M0 at all three sampling times.

### 2.3. Integrated Physiological Assessment Supports Selection of a Favorable MT Treatment

**Figure 4:**
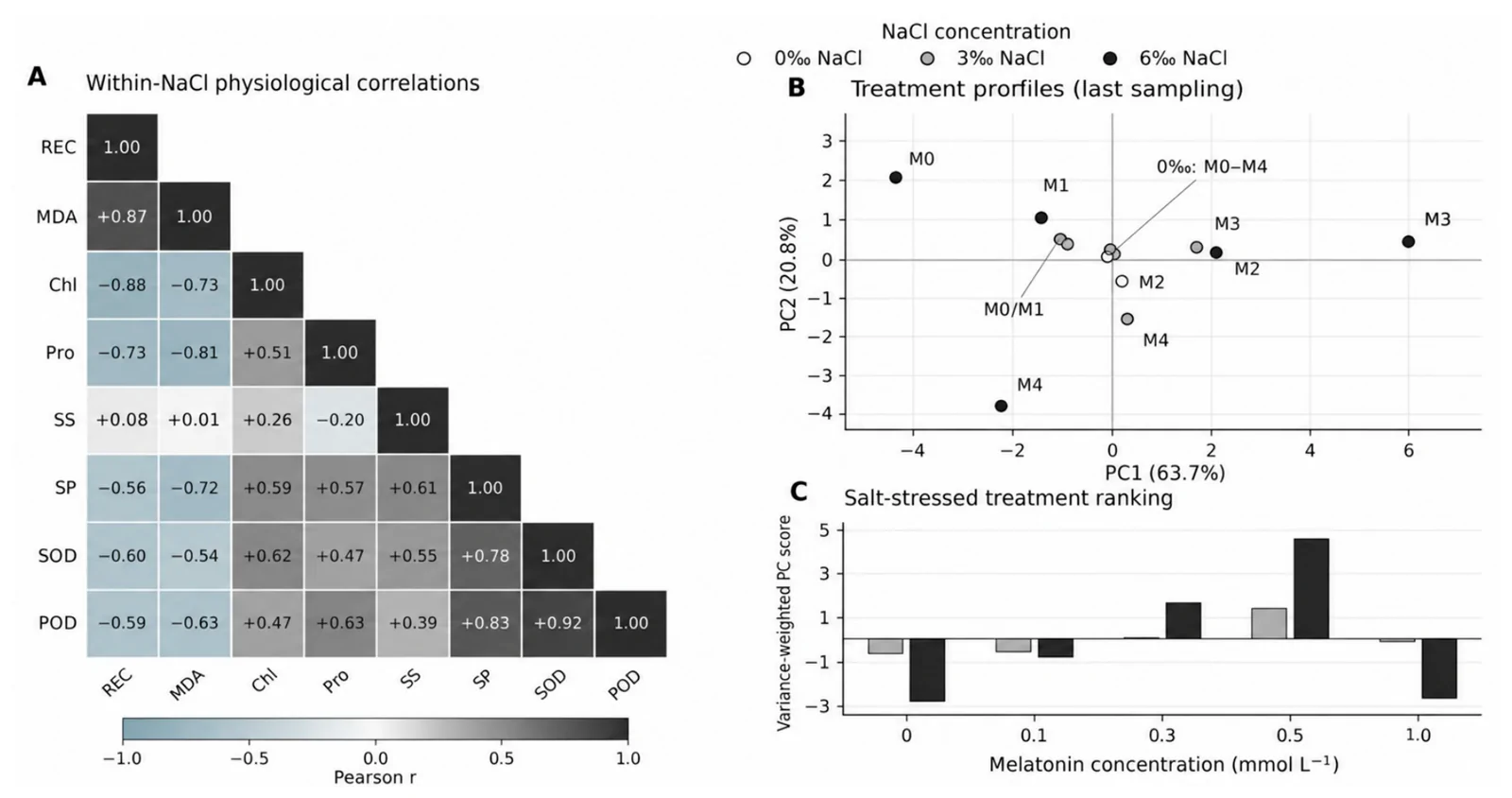
Relationships among physiological traits and integrated responses to exogenous melatonin in leaves of *Salix matsudana* × *S. alba* seedlings under different NaCl concentrations. (**A**) Pearson correlations among eight traits after centering treatment means within each NaCl level; (**B**) principal component analysis of all 15 NaCl × MT treatment combinations; (**C**) variance-weighted PC1–PC2 scores for the two saline treatments. M0–M4 correspond to the MT concentrations shown in panel C. The signs of PC1 and PC2 were oriented so that higher scores corresponded to a lower mean of standardized REC and MDA. All correlation, PCA, and composite-score analyses were based on treatment means and are interpreted descriptively.

To compare overall physiological condition among MT treatments, correlation analysis and PCA were conducted using treatment means for eight physiological traits after centering within each NaCl level; PCA additionally used standardization across all 15 treatments at each sampling time (Figure 4). At the final sampling, REC and MDA were strongly positively correlated (*r* = 0.87), and both varied in the opposite direction to Chl (Figure 4A). SP, SOD, and POD also showed broadly consistent changes, whereas SS was only weakly correlated with REC and MDA, suggesting that SS alone was insufficient to characterize the overall physiological response to MT under salt stress. Because the correlation analysis and PCA were based on treatment means, these analyses were used to summarize treatment-level multivariate patterns rather than to infer significant differences among biological replicates.

After within-NaCl centering and standardization across treatments, the first two principal components explained 63.7% and 20.8% of the variation, respectively, accounting for 84.5% in total (Figure 4B). PC1 was negatively associated with REC and MDA and generally positively associated with Chl, Pro, SP, SOD, and POD. Under S3, M3 was clearly separated from M0 and M4 along PC1. Although M4 had higher SS, it did not simultaneously maintain lower membrane injury and higher Chl. Separation among MT treatments was smaller under S2, but M3 remained on the side associated with a more favorable overall physiological condition.

Descriptive integrated scores were then calculated using the variance contributions of the first two principal components as weights. At the final sampling, M3 had the highest score under both S2 and S3 (Figure 4C; Table S2). Under S3, M2 ranked second, whereas M4 scored considerably lower than M3. Rankings at the other sampling times showed that M3 generally retained a favorable position under S3, whereas the leading treatment under S2 varied to some extent with sampling time.

Within the salt stress conditions examined, M3 therefore showed a relatively consistent overall physiological advantage, particularly under S3. This assessment provided the physiological basis for transcriptome comparisons of representative treatments sampled on day 34.

### 2.4. Overall Transcriptomic Changes Under Salt Stress and MT Treatment

Based on the physiological responses and integrated assessment, the untreated control CK (S1M0; 0‰ NaCl and 0 mmol L^−1^ MT) and the three S3 treatments (S3M0, S3M2, and S3M3) were selected for transcriptome comparisons. Across the 12 RNA-seq libraries, 83.15 Gb of clean data were obtained, with at least 6.17 Gb per library and Q30 values above 93.99%; mapping rates ranged from 74.48% to 79.71%. Sample-level PCA, pairwise Pearson correlations, and hierarchical clustering were used to assess relationships among the 12 RNA-seq libraries (Figure S1). Within-treatment correlations were generally high (Pearson r = 0.850–0.934), with some variation among individual biological replicates. De novo assembly yielded 125,103 unigenes and 249,888 transcripts, with N50 values of 1109 and 1396 bp, respectively, and BUSCO completeness values of 73.2% and 89.8%. Of the 125,103 unigenes, 80,202 (64.11%) were functionally annotated in at least one database.

**Figure 5:**
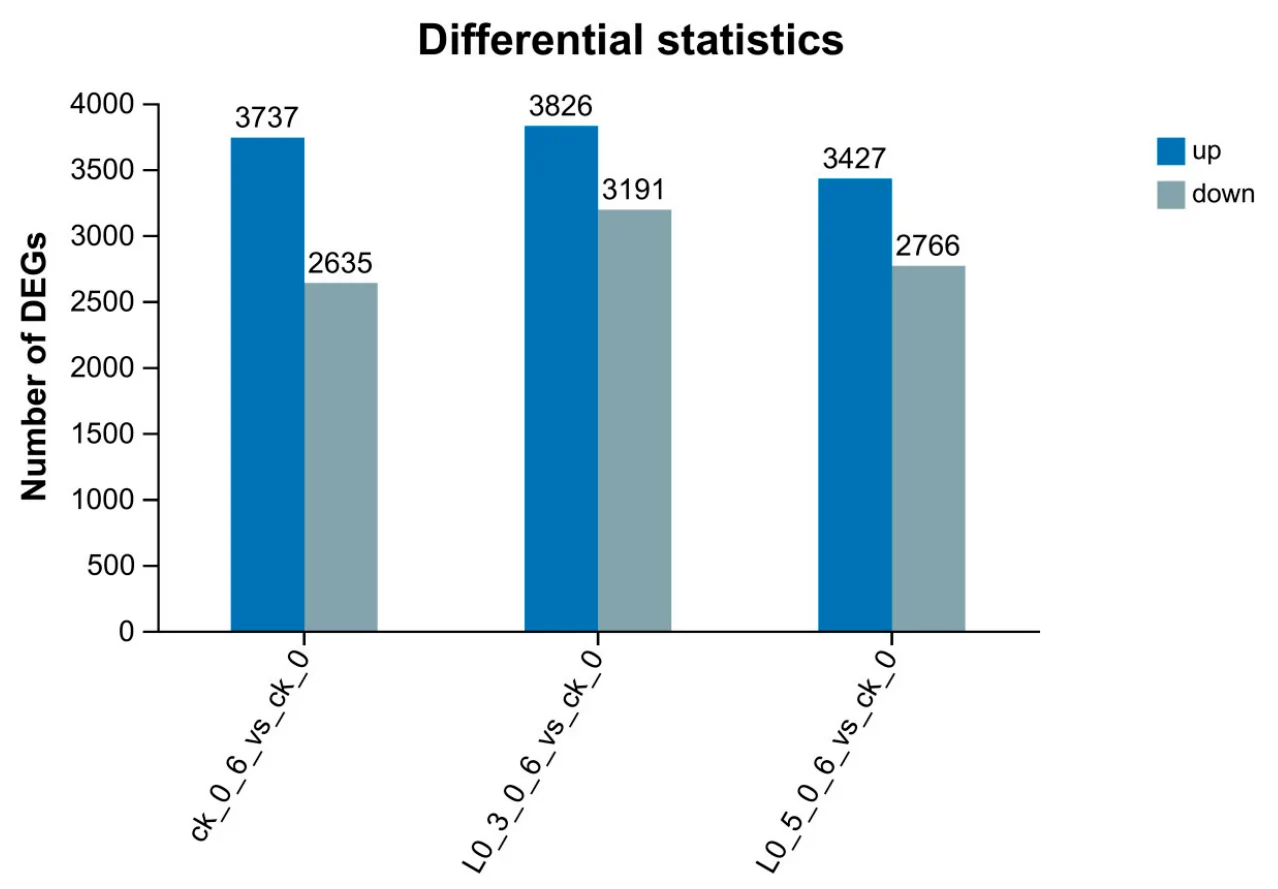
Differential gene expression in leaves of *Salix matsudana* × *S. alba* seedlings under salt stress with or without exogenous melatonin. Bars show the numbers of upregulated and downregulated DEGs in S3M0, S3M2 and S3M3 relative to CK. The original labels ck_0_6, L0_3_0_6 and L0_5_0_6 denote S3M0, S3M2 and S3M3; ck_0 denotes the untreated control.

Relative to CK, 6372, 7017, and 6193 differentially expressed genes (DEGs) were identified in S3M0, S3M2, and S3M3, respectively, with more upregulated genes than downregulated genes in all three comparisons (Figure 5). These common-reference comparisons characterize the transcriptional states of severe salt stress and the two representative MT treatments relative to CK, providing a basis for comparing shared and treatment-specific departures from the untreated state.

**Figure 6:**
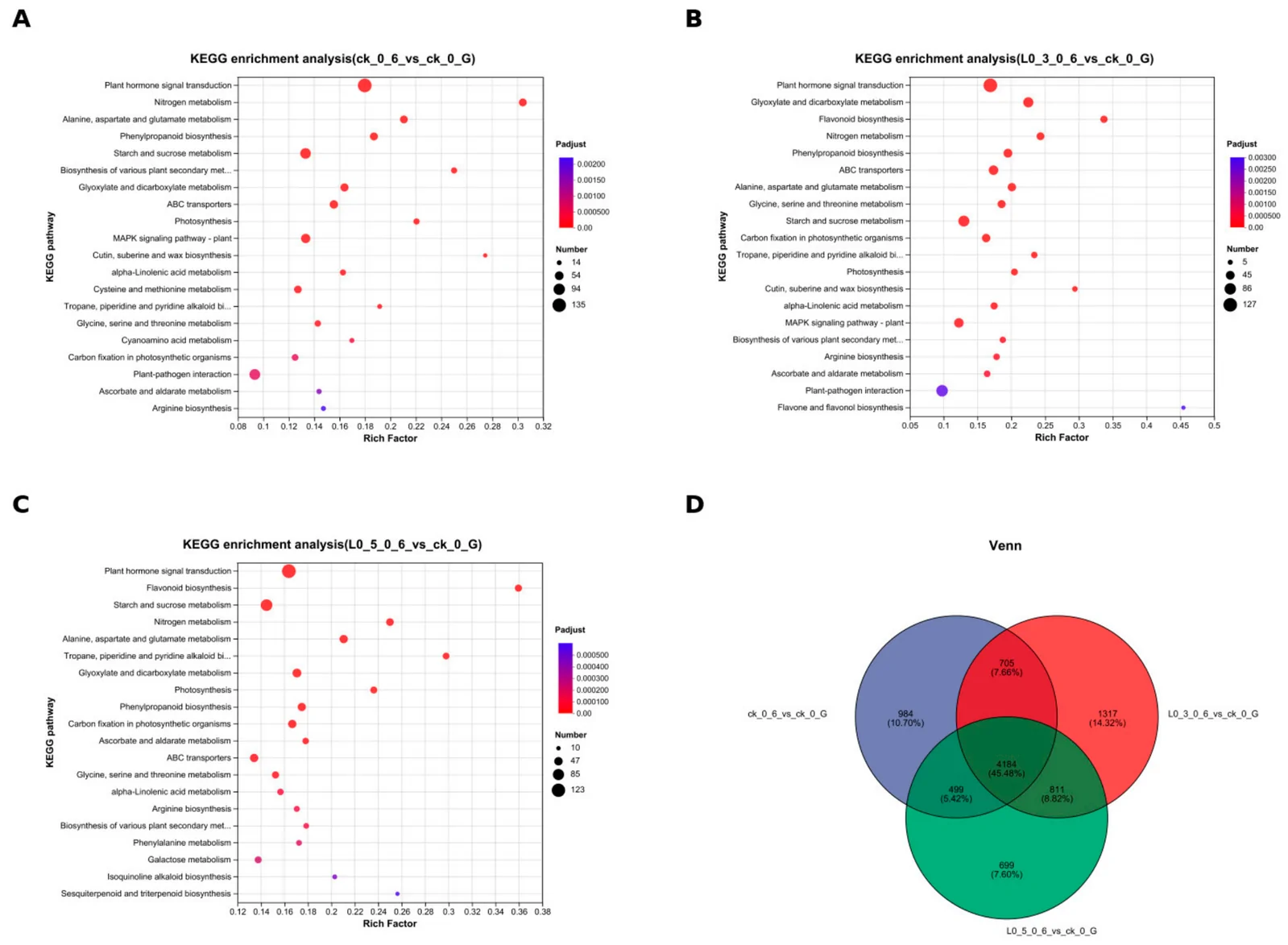
KEGG enrichment and overlap of differentially expressed genes in leaves of *Salix matsudana* × *S. alba* seedlings under salt stress with or without exogenous melatonin. (**A**–**C**) KEGG enrichment of DEGs in S3M0, S3M2 and S3M3 relative to CK, respectively; (**D**) overlap among the three DEG sets. Bubble size represents the number of DEGs, and color indicates the adjusted *p* value.

Among the three comparisons with CK, S3M2 had the most DEGs and S3M3 the fewest. Venn analysis identified 4184 DEGs shared by all three comparisons, while 984, 1317, and 699 DEGs were unique to the S3M0, S3M2, and S3M3 comparisons with CK, respectively (Figure 6D). Thus, the treatments shared many expression differences relative to CK but also had distinct DEG sets.

Gene Ontology (GO) classification assigned DEGs from the three comparisons to similar functional categories (Figure S2). Within the biological process domain, cellular process and metabolic process were the predominant categories. Membrane part and cell part contained many DEGs in the cellular component domain, whereas binding and catalytic activity predominated in the molecular function domain. Gene numbers differed among categories across comparisons, but the major functional categories were broadly consistent.

Kyoto Encyclopedia of Genes and Genomes (KEGG) enrichment identified plant hormone signal transduction, starch and sucrose metabolism, nitrogen metabolism, and phenylpropanoid biosynthesis among the displayed enriched pathways in all three CK-referenced comparisons (Figure 6A–C). Flavonoid biosynthesis was displayed in S3M2 and S3M3, whereas MAPK signaling and cutin, suberine and wax biosynthesis were displayed in S3M0 and S3M2 but not among the top 20 pathways shown for S3M3. Subsequent analysis therefore focused on expression changes in genes associated with hormone signaling and carbohydrate metabolism.

### 2.5. Expression Patterns of Genes Associated with Hormone Signaling and Sugar Metabolism

**Figure 7:**
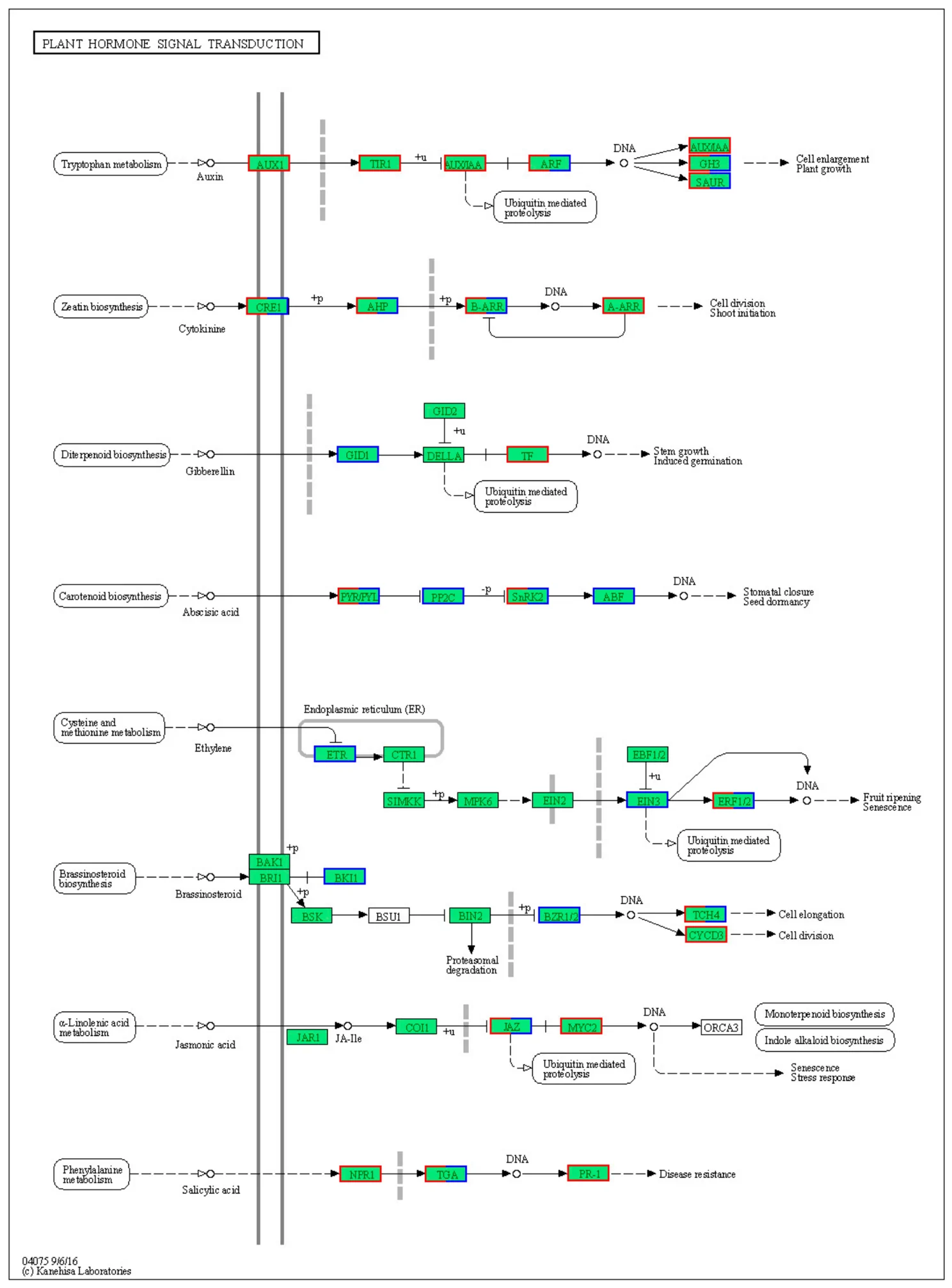
Expression patterns of genes mapped to the plant hormone signal transduction pathway in leaves of *Salix matsudana* × *S. alba* seedlings. Expression changes are shown for S3M3 (6‰ NaCl + 0.5 mmol L^−1^ MT) relative to CK (no NaCl or MT). Green boxes indicate mapped pathway components. Red and blue outlines denote components containing upregulated and downregulated genes, respectively.

Based on the enrichment results, expression differences in key pathways were examined between S3M3 and CK. Because this contrast includes both salt stress and MT treatment relative to CK, the resulting pathway patterns are interpreted as features of the S3M3 treatment state rather than as MT-specific transcriptional effects. A total of 123 DEGs were mapped to the plant hormone signal transduction pathway, with similar numbers of upregulated and downregulated genes (Figure 7). Among auxin signaling-related genes, eight *AUX*/*IAA* members were upregulated, whereas the *SAUR* family included both upregulated and downregulated members. Among abscisic acid (ABA) signaling-related genes, 22 *PP2C*-related and 13 *ABF*-related genes were downregulated, while *PYL* members changed in different directions. These results revealed diverse expression responses among hormone signaling-related gene families and their members.

**Figure 8:**
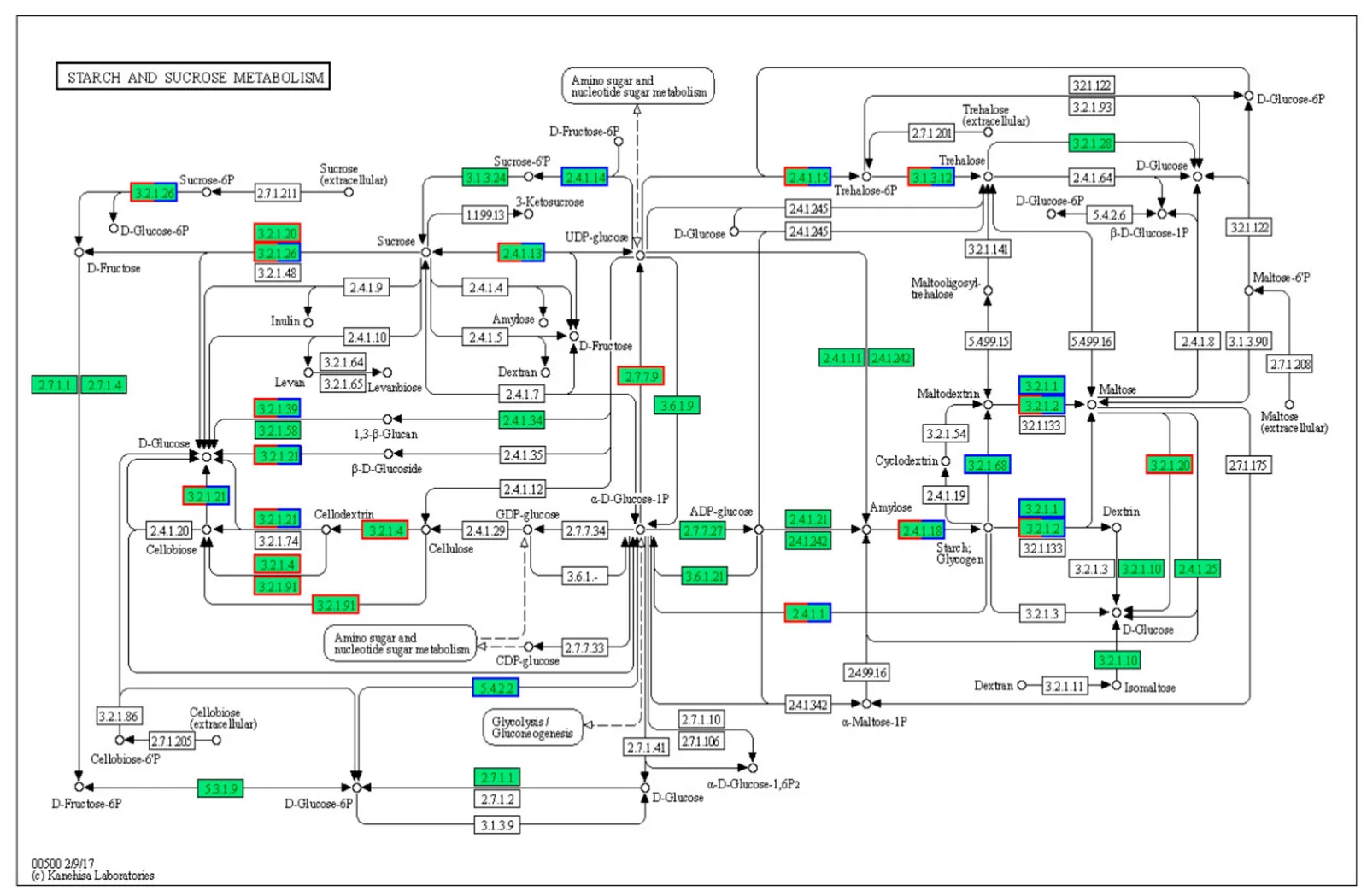
Expression patterns of genes mapped to starch and sucrose metabolism in leaves of *Salix matsudana* × *S. alba* seedlings. Expression changes are shown for S3M3 relative to CK. Green boxes indicate mapped pathway components. Red and blue outlines denote components containing upregulated and downregulated genes, respectively.

In the same comparison, 88 DEGs were mapped to starch and sucrose metabolism, with more downregulated than upregulated genes (Figure 8). Genes associated with trehalose-phosphate synthase (TPS) were predominantly downregulated, with 19 members downregulated and two upregulated. Amylase- and isoamylase (ISA)-related genes were also downregulated, whereas some β-glucosidase-related genes were upregulated. These differences involved several steps in sugar metabolism, with relatively consistent directions of change among TPS-related and some starch metabolism-related genes.

In addition, TF annotation identified 1439 candidate TF sequences belonging to 33 families, including MYB, AP2/ERF, WRKY, NAC, bHLH, bZIP, and GRAS.

### 2.6. qRT-PCR Validation of Candidate Gene Expression Patterns

**Figure 9:**
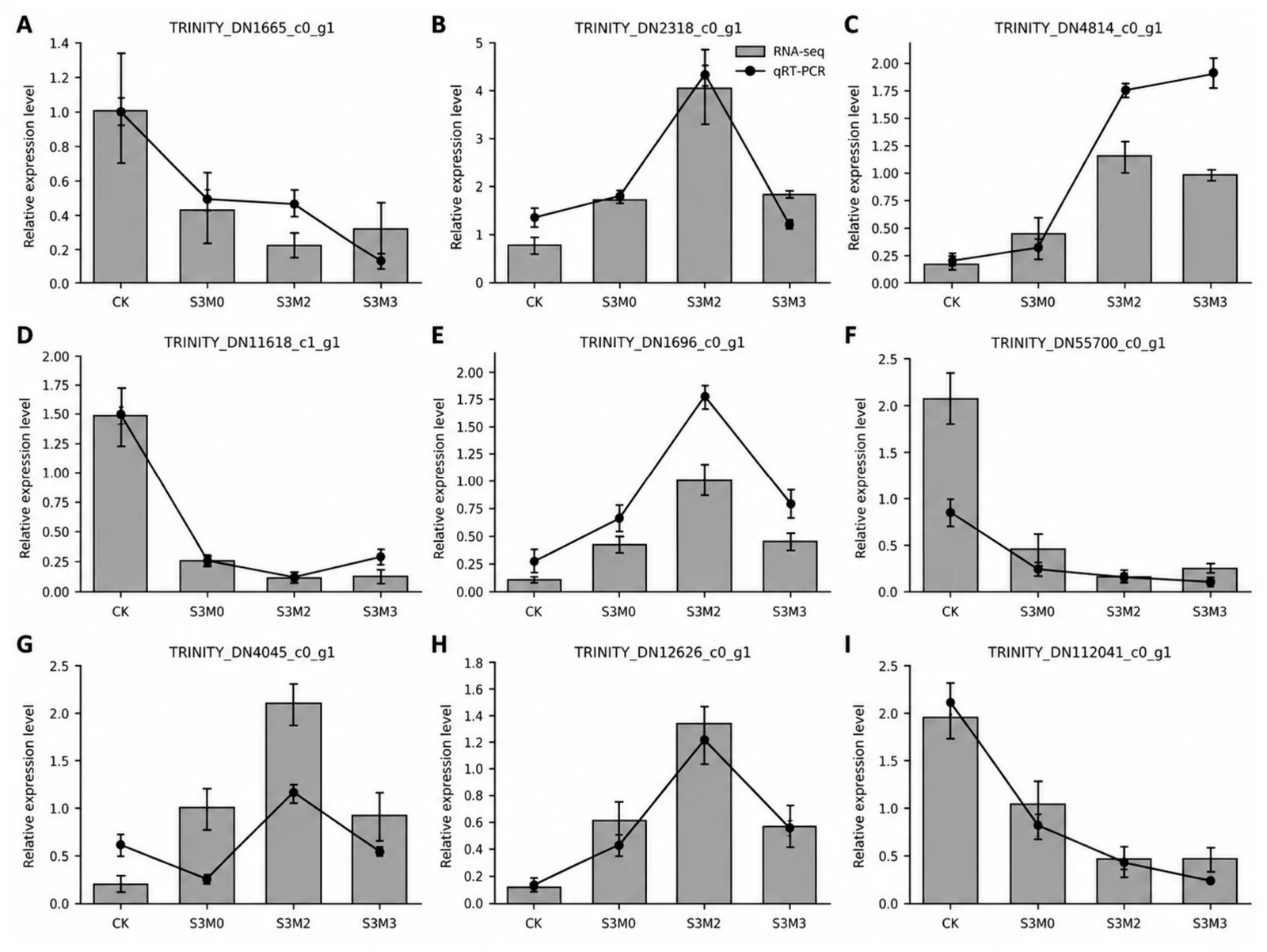
Comparison of RNA-seq and qRT-PCR expression patterns of nine candidate genes in leaves of *Salix matsudana* × *S. alba* seedlings under salt and melatonin treatments. (**A**–**I**) TRINITY_DN1665_c0_g1, TRINITY_DN2318_c0_g1, TRINITY_DN4814_c0_g1, TRINITY_DN11618_c1_g1, TRINITY_DN1696_c0_g1, TRINITY_DN55700_c0_g1, TRINITY_DN4045_c0_g1, TRINITY_DN12626_c0_g1 and TRINITY_DN112041_c0_g1, respectively. Gray bars represent RNA-seq expression values (means ± SD of three biological replicates), whereas black lines represent qRT-PCR results. CK represents the untreated control.

Nine candidate genes were analyzed by quantitative reverse transcription PCR (qRT-PCR) to assess their RNA-seq expression trends in CK, S3M0, S3M2, and S3M3 (Figure 9). The Trinity identifiers, available NR-based putative annotations, primer sequences, and predicted amplicon lengths of the nine candidates are provided in Table S3. Overall, the main trends were broadly consistent between the two methods. TRINITY_DN1665_c0_g1, TRINITY_DN11618_c1_g1, TRINITY_DN55700_c0_g1, and TRINITY_DN112041_c0_g1 showed lower expression in S3M0, S3M2, and S3M3 than in CK (Figure 9A,D,F,I).

By contrast, TRINITY_DN4814_c0_g1, TRINITY_DN1696_c0_g1, and TRINITY_DN12626_c0_g1 showed higher expression under MT treatment (Figure 9C,E,H). Both methods indicated that TRINITY_DN1696_c0_g1 and TRINITY_DN12626_c0_g1 had their highest expression in S3M2, followed by a decrease in S3M3. TRINITY_DN2318_c0_g1 and TRINITY_DN4045_c0_g1 also showed higher expression in S3M2 (Figure 9B,G), indicating that candidate genes differed in their responses to MT concentration.

The magnitude of expression changes and some treatment rankings differed between qRT-PCR and RNA-seq, particularly for TRINITY_DN2318_c0_g1 in S3M3 and TRINITY_DN4045_c0_g1 in S3M0. Accordingly, the qRT-PCR results supported the directional trends of several selected candidates while also revealing gene-specific differences in the magnitude of change and treatment ranking between the two measurement approaches.

### 2.7. A Hypothesis-Generating Working Model of Physiological and Transcriptional Responses

**Figure 10:**
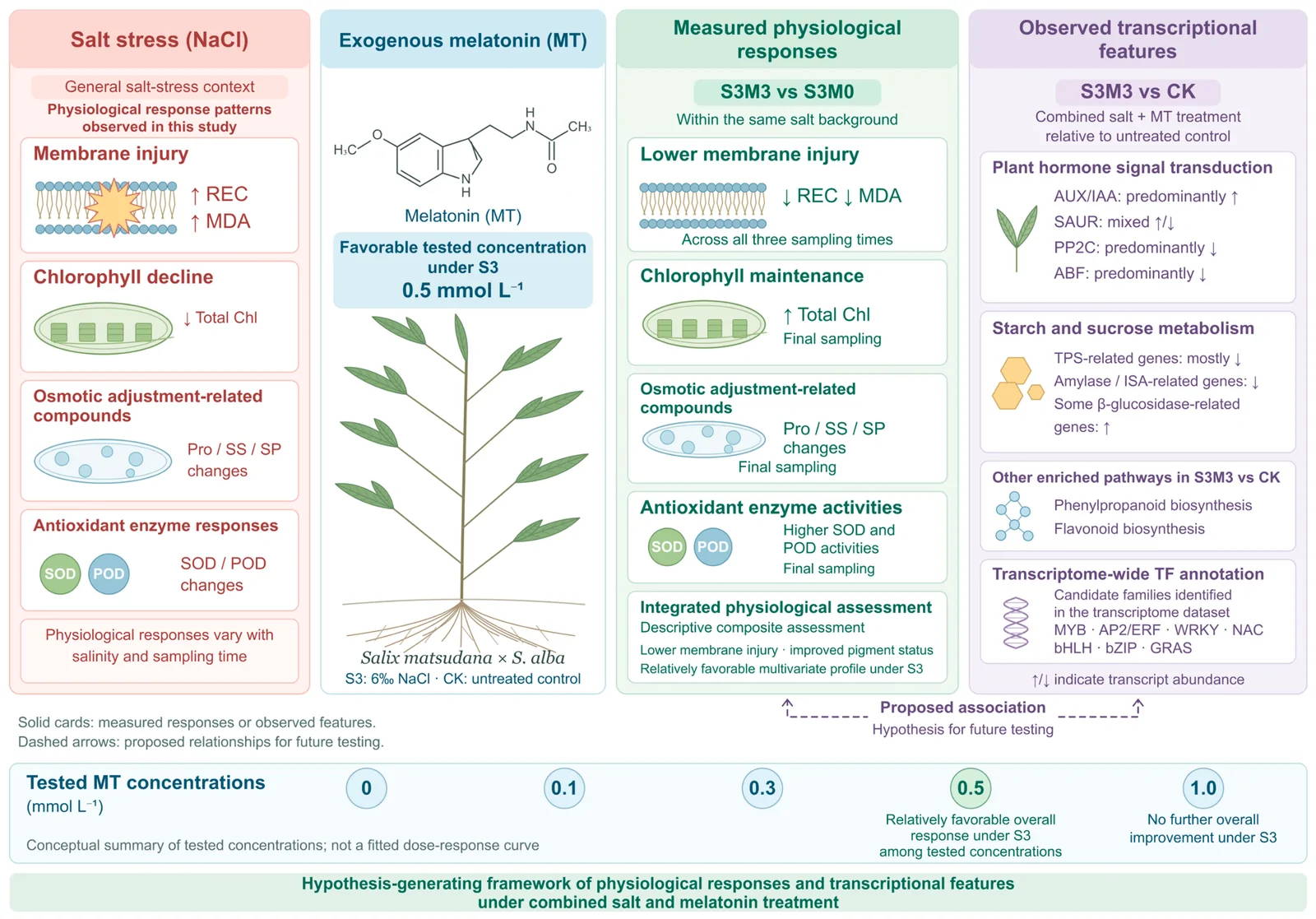
Hypothesis-generating conceptual model integrating physiological responses (S3M3 vs. S3M0) and transcriptional features (S3M3 vs. CK) in *Salix matsudana × S. alba.* Dashed arrows indicate proposed associations.

A hypothesis-generating conceptual model was developed to summarize the measured physiological responses, transcriptional features observed relative to CK, and proposed relationships between these two levels of evidence (Figure 10). The physiological responses to salt stress were characterized by higher REC and MDA content and lower total chlorophyll content. An appropriate MT treatment was associated with reduced membrane injury, chlorophyll maintenance, and changes in osmotic adjustment-related compounds and antioxidant enzyme activities. Compared with M0 at the final sampling under S3, M3 showed lower REC, lower MDA content, higher Chl, Pro, SS, and SP contents, and higher SOD and POD activities. However, responses differed with NaCl level and sampling stage, and the higher SS in M4 did not accompany a better overall physiological condition. Direct physiological comparisons, summarized by the composite assessment, supported M3 as a favorable treatment among the concentrations tested, but this finding cannot be directly generalized to all salinity levels and growth stages.

The transcriptional component of the model mainly involves plant hormone signal transduction and starch and sucrose metabolism. Expression differences between S3M3 and CK in *AUX*/*IAA*, *SAUR*, *PP2C*, and *ABF* members, as well as TPS-related and some starch metabolism-related genes, suggest that these processes may contribute to responses under combined salt and MT treatment. The model proposes possible links between these transcriptional changes and reduced membrane injury, Chl maintenance, osmotic adjustment-related compounds, and antioxidant enzyme activities.

## 3. Discussion

### 3.1. Salt Stress Alleviation by MT Depends on Concentration and Environmental Conditions

This study showed that salt stress alleviation by MT in *Salix matsudana* × *S. alba* did not continually improve as the applied concentration increased. Under S3, M3 consistently reduced membrane injury across sampling stages. Compared with M3, M4 resulted in higher REC and MDA content and did not further improve total chlorophyll content at the final sampling (Figure 1). Similar concentration effects have been reported in other plants. Li et al. found that 150 μmol L^−1^ MT improved photosynthesis and biomass in salt-stressed watermelon more effectively than 50 or 500 μmol L^−1^ MT [30]. A study of the shrub *Reaumuria trigyna* found that 300 μmol L^−1^ MT provided relatively effective alleviation of alkaline salt stress among the concentrations examined [31]. These findings indicate that an appropriate MT concentration must be established by treatment comparisons; benefits at a lower concentration do not imply that a higher concentration will be more effective. Appropriate concentrations also vary among plant species and experimental conditions, so selection for *Salix matsudana* × *S. alba* should be based on its own physiological responses and application conditions.

Salt stress intensity further influenced the effects of MT. Under S2, the improvements in MDA and Chl in M3 relative to M0 were not significant. Under S3, however, M3 significantly reduced REC and MDA at all three sampling times, while a significant increase in Chl appeared only at the later stage (Figure 1). This pattern agrees with the NaCl × MT interactions detected for REC and MDA and indicates that MT effects at different NaCl levels should not simply be pooled. More severe salt stress may make leaf injury and treatment-related differences more apparent. Moreover, membrane injury, pigment status, and enzyme activities did not change synchronously across stages. An appropriate treatment should therefore be identified by considering several traits and sampling stages rather than the largest change in one trait at a single time.

The MT treatments under S1 provide a reference for interpreting the effects of high concentrations. M4 increased REC without significantly increasing MDA, and its significant reduction in Chl occurred at the first two sampling times (Figure 1), suggesting that high MT concentrations may adversely affect membrane permeability and some physiological processes. Wang et al. reported that high MT concentrations inhibited leaf development in *Arabidopsis* by affecting cell proliferation and endoreduplication [32], indicating that inhibitory effects can also occur under non-stress conditions. Considering both saline and non-saline treatments, M3 can be regarded as an appropriate treatment among the concentrations compared here, with particularly consistent performance under S3.

### 3.2. Links Between Reduced Membrane Injury, Osmotic Adjustment, and Antioxidant Responses

The advantage of an appropriate MT treatment was supported by several convergent physiological changes. Under S3, M3 reduced both REC and MDA and maintained higher Chl at the final sampling (Figure 1), indicating improved membrane integrity and photosynthetic pigment status. REC and MDA varied in the same direction and both varied inversely with Chl (Figure 4), further supporting an association between leaf injury and pigment maintenance. Similarly, MT reduced salt-induced oxidative injury and maintained chlorophyll and net photosynthetic rate in *M. hupehensis* [22], while work in watermelon linked reduced membrane injury to improved photosynthetic and redox status [30].

Changes in antioxidant enzyme activities may have contributed to this protection. At the later stage under S3, higher SOD and POD activities in M3 coincided with lower REC and MDA (Figure 1 and Figure 3), indicating coordinated changes in antioxidant enzyme activities and membrane-injury markers. Because ROS levels were not directly quantified, these responses are interpreted as indirect physiological evidence rather than direct demonstration of reduced ROS accumulation. Research in tomato has shown that MT can enhance antioxidant enzymes and the ascorbate–glutathione cycle while reducing ROS accumulation and membrane injury [24]. However, higher enzyme activities did not always correspond to a better physiological condition in the present study. For example, M4 retained relatively high enzyme activities under some saline treatments without equivalent membrane protection. Higher activity may therefore indicate effective defense, but it may also reflect continued exposure to substantial stress. Direct measurements of H_2_O_2_, ${O_{2}}^{-}$, and total ROS, together with catalase (CAT) activity, would further clarify the relationship between antioxidant enzyme responses and oxidative status.

Changes in Pro and SP suggest that MT also affected processes associated with osmotic adjustment and metabolic maintenance. Under S3, M3 increased Pro and maintained higher SP at the final sampling (Figure 2). Together with reduced membrane injury, these changes formed a more favorable physiological profile. In tomato, MT promoted Pro accumulation together with increased activities of enzymes involved in its synthesis [24]. Genetic studies in *Arabidopsis* have shown that both Pro synthesis and catabolism contribute to growth maintenance and redox balance at low water potential, so their roles cannot be represented by total content alone [33]. SP was positively correlated with SOD and POD activities in the analysis (Figure 4A), indicating covariation between soluble-protein status and antioxidant enzyme responses. However, SP represents the bulk soluble-protein pool, whereas the enzyme assays quantify catalytic activity; these correlations therefore do not show that SP accumulation caused higher SOD or POD activity. Accordingly, changes in Pro and SP were interpreted as physiological responses accompanying MT treatment and evaluated together with membrane injury, pigment status, and antioxidant enzyme activities rather than as independent evidence of improved salt tolerance.

The lack of agreement between SS and other traits is a notable finding. At the later stage under S3, M4 had the highest SS but did not simultaneously have the lowest REC and MDA or the highest Chl. At the earlier sampling times, lower membrane injury in M3 was not consistently accompanied by higher SS than in M0 (Figure 1 and Figure 2). The clearer SS accumulation at the final sampling may reflect a time-dependent shift in the balance between sugar production, consumption, and export during continued salt exposure. Higher content may contribute to osmotic protection, but it may also accompany restricted utilization or export. An increase in SS alone is therefore insufficient evidence of salt stress alleviation. Combining injury markers, pigment status, osmotic adjustment-related compounds, and enzyme activities provides a more useful assessment of the overall effects of MT than assuming that a higher value of any single trait is always beneficial.

### 3.3. Transcriptional Changes Related to Hormone Signaling and Sugar Metabolism and Their Physiological Implications

The transcriptomic results provide molecular clues for characterizing treatment-associated transcriptional states. Because S3M0, S3M2, and S3M3 were compared against the same CK reference, these comparisons emphasize shared and distinct departures from the untreated state rather than isolating MT-specific transcriptional effects. Plant hormone signal transduction and starch and sucrose metabolism were prominent in the enrichment analysis (Figure 6), consistent with findings in alfalfa linking MT treatment to hormone signaling, carbohydrate metabolism, and associated physiological changes [25]. This directly relevant MT–salt stress study provides comparative support for the involvement of hormone signaling and carbohydrate metabolism, whereas the corresponding pathways identified in the present hybrid willow study are treated as candidate transcriptional features rather than functionally established mechanisms. Enrichment of nitrogen metabolism provides a candidate context for the Pro and SP responses, while flavonoid biosynthesis identifies a possible secondary-metabolite contribution to stress responses. MAPK signaling and cutin/suberin/wax biosynthesis additionally highlight signaling and surface-barrier processes in the CK-referenced comparisons. These pathway associations suggest targets for further investigation rather than demonstrating changes in nitrogen-assimilation rates, flavonoid contents, MAPK activity, or surface-barrier deposition.

Expression changes within hormone pathways must be interpreted in light of component functions. Upregulation of *AUX*/*IAA* members and mixed responses among *SAUR* members (Figure 7) suggest adjustments in auxin-related transcriptional responses. Experimental evidence shows that Aux/IAA proteins act as transcriptional repressors and that their TIR1-mediated degradation is an important step in auxin responses [34]. Higher transcript abundance therefore does not necessarily indicate stronger signaling output. A similar distinction is needed for ABA-related components. *PP2C-* and *ABF-*related genes were predominantly downregulated in this study. In the core ABA signaling system, clade A PP2Cs inhibit SnRK2 activity, whereas ABFs contribute to downstream transcriptional output [35]. The downregulation of *PP2C-* and *ABF-*related transcripts indicates adjustments in hormone-response components under combined salt and MT treatment, rather than uniform activation or inhibition of the entire pathway. These expression patterns therefore identify hormone-response components associated with the combined treatment, while their relationship with IAA and ABA abundance or signaling output remains to be further resolved by direct hormone measurements. Accordingly, pathway enrichment and transcript abundance are used here to identify candidate processes associated with the combined treatment rather than to infer pathway activation or metabolic flux.

Sugar metabolism provides an important connection between signaling changes and osmotic adjustment-related compounds. Using mutants and carbon isotope tracing, an *Arabidopsis* study showed that ABA can influence starch degradation through AREB/ABF–SnRK2-related processes and alter carbon export to roots and osmotic adjustment-related compound accumulation under stress [36]. Amylase- and ISA-related genes were downregulated in S3M3 relative to CK (Figure 8), whereas the physiological analysis of SS mainly compared MT treatments with M0 at the same NaCl level. The current findings indicate that several steps in sugar metabolism were involved in responses to combined salt and MT treatment. These physiological and transcriptomic results represent complementary aspects of carbohydrate-related responses but were derived from different treatment contrasts. Soluble sugar content was compared among MT treatments within S3, whereas the transcriptional patterns in Figure 8 represent S3M3 relative to CK. Accordingly, the transcriptional changes in amylase-, ISA-, and TPS-related genes are interpreted as features of carbohydrate metabolism under combined salt and MT treatment rather than as a direct explanation for total soluble sugar accumulation. Further resolution of this relationship would benefit from measurements of individual sugar fractions, starch content, and related enzyme activities.

A study combining molecular simulations and in vitro phosphorylation assays found that trehalose 6-phosphate (T6P) can bind KIN10, the catalytic subunit of SnRK1, and limit its activation, providing direct evidence of a link between sugar status and metabolic regulation [37].

The TF and candidate gene results further illustrate differences among responses. Research in banana has linked MT treatment to expression changes in members of the MYB, NAC, bHLH, and WRKY families [26]. In grapevine, promoter-binding and gene-suppression experiments provided functional evidence for the involvement of MYB108A in MT-associated salt tolerance [38]. By contrast, the MYB, AP2/ERF, WRKY, and other TF families identified in the present study are considered candidates for further functional screening. Similarly, functional analyses in maize showed that ZmMYB33 directly activated ZmF3H6 and that genetic manipulation of ZmF3H6 altered salt tolerance, further illustrating the value of functional validation for candidate stress-responsive regulators [39]. qRT-PCR and RNA-seq supported the main expression trends of some candidates, but the higher expression of TRINITY_DN1696_c0_g1 and TRINITY_DN12626_c0_g1 in S3M2 did not correspond to the highest integrated physiological score (Figure 4 and Figure 9). A peak in the expression of an individual gene therefore need not coincide with the strongest overall protection. Its significance should be interpreted in relation to function, response timing, and treatment comparisons, rather than expression rank alone. A similar distinction applies at the transcriptome-wide level. S3M2 showed more DEGs relative to CK than S3M3, indicating a greater transcriptional departure from the untreated state, whereas S3M3 was associated with the more favorable integrated physiological profile. Thus, the magnitude of transcriptional reprogramming alone should not be considered a measure of treatment effectiveness, and the selection of M3 was based on the convergence of multiple physiological responses rather than on the number or magnitude of transcriptional changes.

The integrated model summarizes the favorable physiological responses together with transcriptional features involving hormone signaling and carbohydrate metabolism. These findings suggest possible links between physiological responses and transcriptional changes, but do not establish a causal mechanism. Future work should directly compare MT-treated and untreated seedlings at the same salinity, assess ion and hormone status and carbohydrate metabolism, and validate candidate genes. Growth and biomass measurements, together with field trials, are needed to evaluate whether the favorable physiological responses to 0.5 mmol L^−1^ MT extend beyond the tested greenhouse conditions.

## 4. Materials and Methods

### 4.1. Plant Material and Experimental Treatments

The experiment was conducted in a greenhouse at the Baima Campus of Nanjing Forestry University. Healthy, uniformly growing one-year-old rooted cuttings of *Salix matsudana* × *S. alba* were transplanted into pots of the same size. The substrate comprised garden soil mixed with a nutrient substrate containing perlite, peat, vermiculite, and composted straw. Each pot received the same substrate mass. Treatments began after approximately one month of acclimation.

Three NaCl levels (0‰, 3‰, and 6‰, designated S1, S2, and S3) and five MT concentrations (0, 0.1, 0.3, 0.5, and 1.0 mmol L^−1^, designated M0–M4) were combined to form 15 treatments. The MT concentration series included a no-MT control and increasing application levels to assess concentration-dependent responses and whether higher concentrations provided additional physiological benefit. Each treatment comprised three biological replicates, with 10 seedlings per replicate, for a total of 450 seedlings. S1M0 served as the control without NaCl or MT (CK). Before treatment, the mean dry mass of the substrate was determined and used to calculate the amount of NaCl to be added. The NaCl treatment levels were expressed in parts per thousand (‰) on a dry-substrate mass basis, with 0‰, 3‰, and 6‰ corresponding to nominal additions of 0, 3, and 6 g NaCl kg^−1^ dry substrate, respectively. Equal volumes of the corresponding solutions were added to the pots. Trays beneath the pots collected leachate, which was returned to the pots during watering. Soil electrical conductivity was monitored every 7 d as an operational indicator for maintaining the intended salinity treatments, and salt was replenished according to the original experimental protocol when necessary. The reported 0‰, 3‰, and 6‰ levels therefore refer to nominal NaCl addition rates based on dry substrate mass rather than measured root-zone EC values. For each treatment, leaves collected from the 10 seedlings within each biological replicate were pooled to form one composite leaf sample, resulting in three independent biological samples per treatment for physiological measurements.

Analytical-grade melatonin powder, commercially purchased by our laboratory, was used to prepare solutions at 0, 0.1, 0.3, 0.5, and 1.0 mmol L^−1^. MT was applied by foliar spraying from the first day of salt treatment, once every 2 d for a total of seven applications, until the solution began to drip naturally from the leaves. Leaves at comparable positions were sampled on days 20, 27, and 34 to measure membrane injury, Chl, osmotic adjustment-related compounds, and antioxidant enzyme activities.

### 4.2. Leaf Physiological Measurements

REC was calculated as the percentage ratio of blank-corrected conductivity measured after 12 h at 25 °C to that measured after boiling for 10 min and cooling. MDA content was determined by the thiobarbituric acid assay at 450, 532, and 600 nm and expressed as nmol g^−1^ FW. Chl content was calculated as the sum of chlorophyll a and b after extraction with 80% acetone and absorbance measurements at 663 and 645 nm, and expressed as mg g^−1^ FW.

Pro content was determined by the ninhydrin assay at 370 nm and expressed as μg g^−1^ FW. SS and SP contents were measured using the anthrone [40] and Coomassie Brilliant Blue G-250 [41] methods, respectively, and expressed as μg g^−1^ sample mass.

SOD activity was assayed by inhibition of nitroblue tetrazolium photoreduction [42], with one unit defined as the amount of enzyme causing 50% inhibition. POD activity was measured by the guaiacol assay at 470 nm and 30 °C, with one unit defined as an absorbance increase of 0.01 per minute. Both activities were expressed as U g^−1^ leaf mass.

### 4.3. Transcriptome Sequencing and Bioinformatic Analysis

Based on the physiological responses and integrated assessment, CK was selected as the untreated reference, S3M0 represented the severe salt-stress treatment without MT, and S3M2 and S3M3 represented two MT treatments showing relatively favorable physiological performance under S3, with M3 ranking highest and M2 second at the final sampling. Leaves from these four treatments were therefore selected for transcriptome analysis. Three samples per treatment were submitted for sequencing, giving 12 libraries in total. Total RNA was extracted using TRIzol, and genomic DNA was removed with DNase I. RNA degradation was assessed by agarose gel electrophoresis, integrity was evaluated using an Agilent 2100 Bioanalyzer (Agilent Technologies, Santa Clara, CA, USA), and concentration and purity were measured using an ND-2000 (Thermo Fisher Scientific, Wilmington, DE, USA). Poly(A)-tailed mRNA was enriched from 1 μg total RNA using oligo(dT), fragmented, and used to synthesize double-stranded cDNA. Libraries were prepared by end repair, A-tailing, adapter ligation, and fragment selection. After quantification, libraries were sequenced on an Illumina NovaSeq X Plus platform (Illumina, San Diego, CA, USA) to generate 2 × 150 bp paired-end reads.

Raw paired-end reads were trimmed and quality-controlled using fastp (version v0.19.5) with default parameters [43], and the resulting clean reads were assembled de novo using Trinity [44]. Redundant sequences were removed with CD-HIT, and assembly quality was assessed using BUSCO and TransRate. Sequence similarity searches and protein domain searches were performed with DIAMOND and HMMER, with functional annotation against NR, Swiss-Prot, Pfam, orthologous gene cluster, GO, and KEGG databases. Gene abundance was estimated with RSEM [45]; transcripts per million (TPM) were used to describe normalized expression, and read counts were analyzed using DESeq2 [46]. Sample-level relationships were assessed from the normalized expression matrix using principal component analysis (PCA), pairwise Pearson correlation analysis, and hierarchical clustering of the sample-correlation matrix. DEGs were defined by an adjusted *p* value < 0.05 and |log_2_FC| ≥ 1.

S3M0, S3M2, and S3M3 were each compared with CK, and the shared and unique portions of the three DEG sets were examined. Biological process, cellular component, and molecular function categories were summarized from GO annotations. Functional enrichment was performed using GOATOOLS and relevant Python (version 3.12.14) SciPy routines [47]. Based on KEGG enrichment, expression changes were organized for genes in plant hormone signal transduction and starch and sucrose metabolism.

### 4.4. qRT-PCR Analysis of Candidate Genes

Nine candidate transcripts (identifiers in Figure 9) were selected to compare expression changes measured by qRT-PCR and RNA-seq. The treatments were the same as those used for transcriptome analysis: CK, S3M0, S3M2, and S3M3. Total RNA was reverse-transcribed into cDNA, and qRT-PCR was performed using an Analytik Jena qTOWER2.2 real-time PCR system with SYBR Green chemistry.

Primers were designed using Primer Premier 6 and their sequence specificity was evaluated using TBtools (version v0.6738). The qRT-PCR assay was repeated three times, with three reaction wells used for each gene in each run. Relative expression was calculated by the comparative Ct method [48], with ARF8 (locus tag: NC_003074) as the internal reference gene. The Ct value of ARF8 was subtracted from the target-gene Ct value to obtain ΔCt, and the calibrator ΔCt was then subtracted from the sample ΔCt to obtain ΔΔCt. Relative quantity (RQ) was calculated as follows:

RQ = 2^−ΔΔCt^ (1)

### 4.5. Statistical Analysis and Integrated Physiological Assessment

Physiological measurements were analyzed using a full factorial repeated-measures ANOVA, with NaCl (0, 3, and 6‰) and MT concentration as categorical between-unit factors and sampling time (20, 27, and 34 d) as a categorical within-unit factor. The same three biological replicate groups per NaCl × MT treatment were followed across sampling dates. Leaves from 10 seedlings were pooled within each replicate, which constituted one experimental unit. All main effects, two-way interactions, and the three-way interaction were included. Between-unit effects were tested against the replicate-within-treatment error; Greenhouse–Geisser-adjusted degrees of freedom were used for all effects involving time. For each omnibus effect, *p* values across the eight traits were adjusted using the Benjamini–Hochberg procedure, with q < 0.05 defining significance. Secondary MT comparisons within each trait, NaCl level, and sampling date used fitted cell means and the corresponding date-specific residual variance from the full model, with Tukey-adjusted *p* values and simultaneous 95% confidence intervals (five means per family; 30 residual degrees of freedom). Letter groupings apply only within these comparison families. Residual normality and variance heterogeneity were examined. Multivariate repeated-measures tests using Pillai’s trace were used as sensitivity analyses for time-related effects. As an additional multiplicity sensitivity check, BH adjustment was applied across all 56 omnibus tests.

Correlation analysis and PCA used treatment means for REC, MDA, Chl, Pro, SS, SP, SOD, and POD. A matrix of 15 treatments × eight traits was constructed separately for each sampling time. Within each NaCl level, the mean of the five MT treatments was subtracted from each value of a given trait, and Pearson correlations were calculated among the centered traits. For PCA, these centered data were standardized using the standard deviation of each trait across all 15 treatments at that sampling time. Principal component scores and variance contributions were obtained by singular value decomposition. This procedure emphasized MT-associated changes within a NaCl level and prevented differences in means among NaCl levels from dominating the ranking.

An integrated score was calculated as the weighted mean of the PC1 and PC2 scores, using their proportions of explained variance as weights:

(2) $$F=\frac{v_{1}\mathrm{PC}1+v_{2}\mathrm{PC}2}{v_{1}+v_{2}}$$

where v_1_ and v_2_ are the variance contributions of PC1 and PC2, respectively. Component directions were oriented so that increasing scores corresponded to lower mean standardized REC and MDA. Relative MT treatment rankings were compared within each NaCl level and sampling time. PCA was conducted separately for each sampling time, and absolute scores were not compared across times. Physiological statistical analyses were performed in Python using NumPy, SciPy, and statsmodels. Figures were plotted using Matplotlib (version 3.11.2). Finally, physiological changes were integrated with transcriptomic results for the specified comparisons to construct a model of possible physiological and transcriptional links.

## 5. Conclusions

The effects of exogenous MT on salt stress alleviation in *Salix matsudana × S. alba* depended on concentration and salinity. Under 6‰ NaCl, 0.5 mmol L^−1^ MT consistently reduced REC and MDA across three sampling times and improved chlorophyll status, Pro and SP contents, and SOD and POD activities at the final sampling. The integrated physiological assessment supported this concentration as a favorable treatment among those tested, whereas 1.0 mmol L^−1^ provided no further overall improvement. Its beneficial effects were less consistent under 3‰ NaCl. Transcriptomic comparisons with the untreated control identified candidate pathways involving hormone signaling and starch and sucrose metabolism, but did not isolate MT-specific transcriptional effects. These findings support further evaluation of 0.5 mmol L^−1^ MT under the tested greenhouse conditions, while its applicability to other growth stages, salinity conditions, and field environments requires validation.

## Data Availability

The data presented in this study are available on request from the corresponding author.

## Supplementary Materials

The following supporting information can be downloaded at: https://www.mdpi.com/article/10.3390/plants15213304/s1. Figure S1. RNA-seq sample relationships among the 12 libraries. (a) Principal component analysis of sample-level expression profiles; (b) Pearson correlation matrix with hierarchical clustering. Numbers 1–3 indicate biological replicates within each treatment; Figure S2. Gene Ontology (GO) classification of differentially expressed genes in leaves of *Salix matsudana × S. alba* seedlings under salt stress with or without exogenous melatonin; Table S1. Full factorial repeated-measures ANOVA results for the effects of NaCl, melatonin concentration, sampling time, and their interactions on physiological traits; Table S2. Variance-weighted principal component scores and rankings of melatonin treatments under different NaCl levels at different sampling times; Table S3. Available putative annotations of the nine qRT-PCR targets.

## Abbreviations

The following abbreviations are used in this manuscript:

ABA	Abscisic acid
ANOVA	Analysis of variance
Chl	Total chlorophyll
CK	Control without NaCl or MT
DEGs	Differentially expressed genes
FW	Fresh weight
GO	Gene Ontology
HSD	Honestly significant difference
IAA	Indole-3-acetic acid
ISA	Isoamylase
KEGG	Kyoto Encyclopedia of Genes and Genomes
MAPK	Mitogen-activated protein kinase
MDA	Malondialdehyde
MT	Melatonin
NBT	Nitroblue tetrazolium
PCA	Principal component analysis
POD	Peroxidase
Pro	Proline
qRT-PCR	Quantitative reverse transcription polymerase chain reaction
REC	Relative electrolyte conductivity
ROS	Reactive oxygen species
RQ	Relative quantity
SD	Standard deviation
SOD	Superoxide dismutase
SP	Soluble protein
SS	Soluble sugars
T6P	Trehalose 6-phosphate
TFs	Transcription factors
TPM	Transcripts per million
TPS	Trehalose-phosphate synthase
